# Comparing Numerical Comparison Tasks: A Meta-Analysis of the Variability of the Weber Fraction Relative to the Generation Algorithm

**DOI:** 10.3389/fpsyg.2018.01694

**Published:** 2018-09-11

**Authors:** Mathieu Guillaume, Amandine Van Rinsveld

**Affiliations:** ^1^Cognitive Science and Assessment Institute (COSA), University of Luxembourg, Luxembourg, Luxembourg; ^2^Centre for Research in Cognitive Neuroscience (CRCN), Université Libre de Bruxelles, Brussels, Belgium

**Keywords:** Approximate Number System, number sense, meta-analysis, methodology, Weber fraction

## Abstract

Since more than 15 years, researchers have been expressing their interest in evaluating the Approximate Number System (ANS) and its potential influence on cognitive skills involving number processing, such as arithmetic. Although many studies reported significant and predictive relations between ANS and arithmetic abilities, there has recently been an increasing amount of published data that failed to replicate such relationship. Inconsistencies lead many researchers to question the validity of the assessment of the ANS itself. In the current meta-analysis of over 68 experimental studies published between 2004 and 2017, we show that the mean value of the Weber fraction (*w)*, the minimal amount of change in magnitude to detect a difference, is very heterogeneous across the literature. Within young adults, *w* might range from < 10 to more than 60, which is critical for its validity for research and diagnostic purposes. We illustrate here the concern that different methods controlling for non-numerical dimensions lead to substantially variable performance. Nevertheless, studies that referred to the exact same method (e.g., Panamath) showed high consistency among them, which is reassuring. We are thus encouraging researchers only to compare what is comparable and to avoid considering the Weber fraction as an abstract parameter independent from the context. Eventually, we observed that all reported correlation coefficients between the value of *w* and general accuracy were very high. Such result calls into question the relevance of computing and reporting at all the Weber fraction. We are thus in disfavor of the systematic use of the Weber fraction, to discourage any temptation to compare given data to some values of *w* reported from different tasks and generation algorithms.

Over 20 years ago, Dehaene made the hypothesis that Humans possess a *Number Sense*, a biologically determined ability that allows us to represent and manipulate large numerical quantities (Dehaene, 1997). This numerical intuition is largely considered as relying on a cognitive system specifically dedicated to number processing called the *Approximate Number System* (ANS, Feigenson et al., 2004; see also Núñez, 2017; for an interesting terminological criticism). The crucial property of such cognitive system is the scalar variability of numerical approximations: numerical estimates of larger quantities are indeed more variable (Platt and Johnson, 1971; Gallistel and Gelman, 2000). Accordingly, the acuity of numerical discriminative processes handling two amounts is not absolute, but relative to the numerical ratio between the considered quantities (i.e., distinguishing 10 from 20 elements is easier than distinguishing 110 from 120 items). Mental number representations were thus hypothesized to go through a logarithmic compression following the Weber-Fechner law (Dehaene, 2003: but see Cantlon et al., 2009; Cicchini et al., 2014; and Piantadosi, 2016).

In order to assess these logarithmic representations, Piazza et al. (2004) were among the first to characterize performance (as well as brain activity) in a numerical discrimination task with the help of a measure directly related to the Weber-Fechner law, the Weber fraction. The Weber fraction is the ratio between the amount just noticeably different from a given magnitude, and the magnitude itself (*w*, see Stevens, 1957; Van Oeffelen and Vos, 1982). From a psychophysical perspective, the Weber fraction can be defined as the noise constant-proportionality parameter fitting the discrimination behavior during a numerical comparison task (see Barth et al., 2006, Appendix B). As a constant scaling ratio, the Weber fraction has the advantage of explicitly depicting the scalar variability across mental representations, which might fluctuate between individuals (see Whalen et al., 1999). More critically to the purpose of the current meta-analysis, this *w* parameter was heavily popularized in the literature as a direct measure of specific numerical quantity processes by some influential studies (e.g., Pica et al., 2004; Piazza et al., 2004). Subsequently, *w* was widely investigated as an individual property that is only subject to significant developmental changes across the lifespan (Halberda et al., 2012) and to refinement through formal instruction (Piazza et al., 2013). For a given age within a given population, *w* was thus considered as a stable predictor of more complex numerical processing such as math ability (Halberda et al., 2008), as well as a crucial clinical predictor of Mathematical Learning Disability (e.g., Mazzocco et al., 2011).

However, some authors recently questioned the stability of the Weber fraction. Due to the substantial amount of studies that were conducted following Halberda et al. study (2008), there were indeed many reports of failure in observing significant relationship between *w* and math ability (e.g., Price et al., 2012; Gilmore et al., 2013; Sasanguie et al., 2013). This raised some theoretical concerns (e.g., Gebuis et al., 2016; Leibovich et al., 2016; Núñez, 2017), as well as many methodological issues (see Dietrich et al., 2015b; for a review). Among these issues, many studies showed that the assessment of ANS acuity, and the measure of *w* itself, are not independent of interference from low level visual cues that are intrinsically confounded with numerical quantities, and they revealed that *w* is nor consistent nor reliable across different tasks (Clayton et al., 2015, 2018; Bugden and Ansari, 2016; Guillaume et al., 2016). Some authors subsequently argued that the procedure used to generate visual arrays substantially influence participant behavior, and therefore the evaluation of w (Inglis and Gilmore, 2014; Clayton et al., 2015; Smets et al., 2015, 2016). In other words, *the Weber* fraction does not seem to be a stable psychophysiological parameter devoid of context; *w* can in fact be variable within one subject as a function of the task and the stimulus properties (but see Julio-Costa et al., 2015; DeWind and Brannon, 2016; for contradicting evidence).

Inglis and Gilmore (2014) went further by experimentally assessing the validity and the reliability of the Weber fraction in comparison to other measures of ANS acuity. Critically, they claimed that *w* was problematic for many reasons: its distribution was not normal but right-skewed, its test-retest reliability was poorer than every other measure of ANS acuity, and more fundamentally, its value was still affected by the way low level visual cues were manipulated in the task. These results do not support the view that the Weber fraction is an invariable psychophysiological parameter devoid of context. In addition, the authors reported that *w* highly correlated with overall accuracy throughout the task. In other words, *w* was nor more precise nor more informative than general accuracy. The advantages of using this parameter are thus disputable, yet it is commonly used and referred to in the literature as an appropriate tool to compare data sets from different published studies (e.g., in Castronovo and Göbel, 2012; Halberda et al., 2012; Geary et al., 2015; Libertus et al., 2016).

In the current meta-analysis, we aim at verifying whether Weber fractions computed from various numerical comparison tasks are stable and consistent in the literature. If this were the case, then its usage should be preferred to compare datasets from different studies. Alternatively, the observation of substantial heterogeneity in Weber fractions would be worrying for researchers and for clinicians who want to compare performance from a particular sample or from an individual to some *typical* performance.

## Methods

### Article search and inclusion criteria

The current meta-analysis only included peer-reviewed articles written in English and published before January 1st 2018 in any scientific journal. Following these inclusion criteria, we independently searched in the three databases *PsycINFO, PubMed*, and *Web of Science* for the documents that included the whole expression “*Approximate Number System*” in their title, abstract, keyword, or main body. The cross-referencing of the three searches yielded 387 unique references. We refined the search by looking within each document for any mention of the terms “*Weber*” or “*fraction*”. We gathered all matching articles and select the ones that (a) described at least one empirical study conducted on humans with no history of atypical development and that (b) explicitly reported the mean value of the Weber fraction (computed from any non-symbolic comparison task) of their sample(s). Sixty-eight publications were thereby included in the current meta-analysis. They are further referenced in our bibliography with an asterisk. All statistical analyses were conducted on R Studio (R Core Team R., 2016).

We considered two substantial aspects affecting the evaluation of *w* in the current meta-analysis, in order to minimize any potential risk of bias. First, we highlighted each reported *w* as a function of the mean age of the participants. As noted by Halberda et al. (2012), performance–and subsequently *w*–is intrinsically more heterogeneous in children than in adults (see also, Siegler, 2007). It is consequently insufficient to investigate the variability of this measure within young children. For this reason, we decided to focus on young adults to get a clearer picture of the stability of *w* throughout the literature. Such picture is actually critical to support any claim that Weber fractions are reliable and invariable measures of ANS acuity.

Secondly, and more critically for the purpose of the current meta-analysis, the procedure used to generate stimuli–and to control for non-numerical visual cues–does not have a negligible impact on the value of the Weber fraction (Inglis and Gilmore, 2014; Clayton et al., 2015; Smets et al., 2016). In numerical comparison tasks, participants are sensitive to non-numerical dimensions, and they might base their judgments on them (see Gebuis et al., 2016), so that any systematic confound between the number and one visual property substantially affects behavior. In other words, participants might strategically use available visual information to help them to respond to the task (e.g., the larger array is likely to have more elements). Therefore, paradigms that control for various non-numerical cues at the same time lead to worse performance–and thus larger *w*–than methods involving the manipulation of only one dimension (Smets et al., 2015). The values of *w* reported in a given publication are thus not independent of the properties relative to the methodology used to acquire the data (see for instance, Dietrich et al., 2015b). Although we did not aim for the evaluation of specific influence of a given generation algorithm on participants' performance, we decided to emphasized the properties of the task–and their stimuli–that underlay every considered *w*. However, it should be noted that we did not consider any other methodological aspects that may affect performance (such as the duration of stimulus presentation or the range of the displayed numerical quantities, see Clayton et al., 2015; Smets et al., 2016), as they drastically fluctuated from studies to studies and were thus difficult to categorize in such meta-analysis. We describe how we categorize the dataset in the following section.

### Algorithm description and categorization

It is worth noting that a sizeable amount of the retained publications described data collected from more than one participant sample (e.g., comparing different age groups or different methodologies, having different data points in a longitudinal setup). For this reason, we decided to consider data at the sample level, and not at the study level. We then arranged all samples by three categories.

The first category contains the typically developing human samples from publications that explicitly mention the use of the *Panamath*, which is an assessment software freely available at www.panamath.org. *Panamath* is actually the only existing program that can be implemented with the greatest of ease to test participants or patients, and to directly obtain a performance index, as well as the computation of their Weber fraction. It is thus well known among researchers and practitioners interested in evaluating non-symbolic number abilities. Experimental paradigms of all the samples within this category thus share strong similarities due to the use of the same software. This especially includes the display of two arrays of dots with different colors (blue and yellow) at the same time (see Halberda et al., 2008; for further methodological details). Nonetheless, there may still be some dissimilarity between the experimental conditions because *Panamath* allows researchers to modify some stimuli properties at their best convenience, such as the display duration and the maximal array size. It should be noted that these adaptations are primarily intended to account for the potential youth of the subject taking the test. Anyway, we disregarded such slight modifications in the current meta-analysis, and we considered all samples assessed with *Panamath* in one category.

The second category comprises the samples from studies following Dehaene's et al. (unpublished manuscript) recommendations to construct their stimuli (from Piazza et al., 2004). The authors highlighted in their manuscript that some visual properties are inherently confounded with the number of items in an array. For instance, the picture with the largest number of items is expected to occupy the largest area and/or to possess on average the smallest elements. For this reason, they suggested using a generation algorithm designed to maintain constant one visual property across both displayed arrays, so that this dimension could not be informative to make the decision. Typically, such scripts either consider the individual item size (IIS) or the total occupied area (TOA). However, because number (*N*) is the multiplicative factor between these two parameters, such as IIS × N = TOA, the dimension that is not kept constant across the arrays is systematically correlated with number. To overcome this limitation, Dehaene et al. (unpublished manuscript) recommended generating exactly half of the stimuli with one constant dimension, and the other half with the second unvarying parameter, so that a participant that would strategically make use of the information from one non-numerical parameter would obtain the correct answer in only 50% of the case (which is the chance level). In our meta-analysis, we labeled these programs as “One-dimensional” algorithm as they control for one visual dimension at a time. Noticeably, the *Panamath* software follows this creation rule, as it controls for one visual dimension at a time. Yet the item sizes within one given array are not constant in *Panamath*. We thus excluded this script from the second category and we only considered here studies following the half IIS/half TOA constant rule from Dehaene et al. (unpublished manuscript), without any further restriction.

In the third and last category, we considered all other studies that did not use any of the previously described generation scripts. It is noteworthy that none of these manuscripts put aside the methodological concern that many visual dimensions are inherently confounded with number. On the contrary, their generation procedures all featured their own consideration for controlling for more than one visual parameter at a time (besides IIS and TOA, such as the length of the convex hull formed by the array or the item density). Among these procedures, one could refer to Gebuis and Reynvoet's (2011) program that manipulates the congruity (or incongruity) of five different dimensions with number throughout the stimuli, to the paradigm of Mussolin et al. (2012) that used collections with various elements richer than single dots, and to the paradigm of DeWind et al. (2015) that disentangles the relative contribution of three orthogonal dimensions (number, spacing, and size) within participants' performance. As these methods accounted for more than one-dimension at a time, we labeled these “Multi-dimensional” algorithms.

## Results

### Description of the considered samples

Within the 68 scientific publications that were considered in the current meta-analysis, we retained 115 samples of typically developing humans. Nineteen documents that together described data from 28 samples explicitly mentioned using the *Panamath*. Thirty-six articles were included in the “One-dimensional” category, for a total of 63 typical samples that used such generation algorithm. The third category contained 15 documents that reported on 24 typical samples that used “Multi-dimensional” programs. Descriptive data of considered documents and samples are indicated in Table 1. The whole list is available in Supplementary Table S1. Overall, mean sample age was 14.5 years, 95% *CI* [12.5, 16.5], and mean Weber fraction value was 0.30, 95% *CI* [0.27, 0.34]. Two one-way analyses of variance–with Generation algorithm as group factor–revealed that both mean sample age and mean Weber fraction did not significantly differ between the three generation algorithms, both *Fs*_(2, 112)_ < 1. We finally conducted an ANCOVA on the value of *w* controlling for mean age, with Generation algorithm as the group factor; this analysis did not lead to any significant effect, *F*_(3, 111)_ < 1.

**Table 1:** Description of the data, as a function of the generation algorithm.

**Generation algorithm**	**Count of documents**	**Count of samples**	**Count of participants**	**Mean sample age (*SD*)**	**Mean weber fraction (*SD*)**
Panamath	19	28	1975	12.9 years (*11.7*)	0.29 (*0.17*)
One-dimensional	36	63	5230	15.1 years (*10.9*)	0.30 (*0.18*)
Multi-dimensional	15	24	882	14.8 years (*9.21*)	0.33 (*0.16*)
Total	70^a^	115	8087	14.5 years (*10.7*)	0.30 (*0.17*)

a *One manuscript (Smets et al., 2016) contained two studies that used two different generation algorithms, one one-dimensional and one multi-dimensional. Another document (Smets et al., 2015) reported on two one-dimensional conditions and one multi-dimensional condition. They are thus considered twice in the count of documents in Table 1. Values in italic (and in brackets) are Standard Deviations, which is specified in the column title by their common acronym as (SD)*.

### Weber fractions in adults

**Figure 1:**
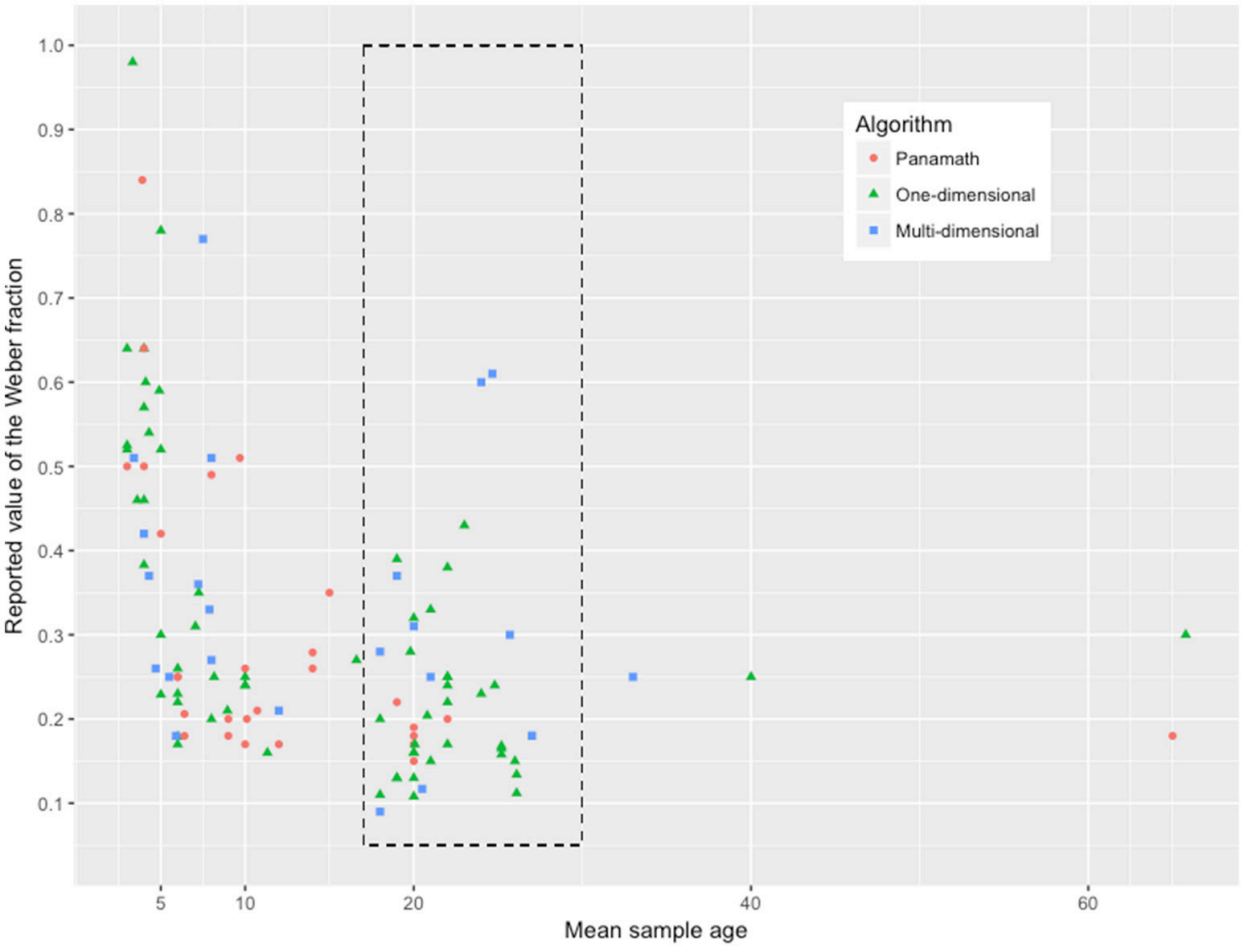
Values of the Weber fraction (from 115 typical samples) as a function of mean sample age. We here distinguish Weber fractions depending on the algorithm that was used to measure them (red dot: Panamath; green triangle: One-dimensional algorithm; blue square: Multi-dimensional program). The dashed rectangle encompasses the values from typical adult participants (aged from 18 to 30 years old), which we further consider in Figure 2.

Despite the overall absence of a significant difference between the three algorithm categories in terms of Weber fractions, a closer look at Figure 1 revealed that *w* means were not totally independent from mean sample age. Pearson correlation coefficient between the two variables was at *r* = −0.41, which was indeed significant, *t*_(113)_ = −4.662, *p* < 0.001. The value of the Weber fraction thus diminished when age increased, which was in line with previous findings that the noisier numerical acuity at younger age is going through some developmental changes and gradually refines over the years (until ~30 years, Halberda et al., 2012). Moreover, due to the inherent variability of data collected in children (Siegler, 2007), we focused our further analyses on samples of adults ranging from 18 to 30 years, in order to be able to compare similar data.

**Figure 2:**
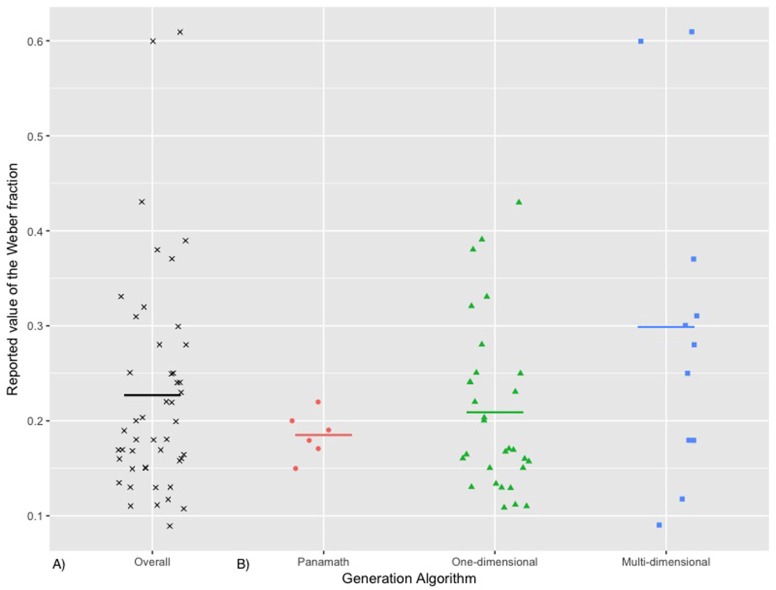
**(A)** Values of the Weber fraction (from 47 samples of typical adults). **(B)** Values of the Weber fraction as a function of the algorithm that was used to measure these values (red dot: Panamath; green triangle: One-dimensional algorithm; blue square: Multi-dimension program). The horizontal lines depict the mean values.

Mean Weber fractions from these selected samples are depicted in Figure 2A. Data was collected from 34 documents comprising 47 typically developed adult samples. Mean sample age was 21.68, 95% *CI* [20.89, 22.46], and mean sample size was 48, 95% *CI* [35, 60]. Critically, the mean Weber fraction was 0.22, 95% *CI* [0.19, 0.26]. The latter value drastically ranged, from a minimal value of 0.09 (“congruent condition” from Smets et al., 2015) to a maximal value of 0.61 (in Dietrich et al., 2016). Even in young adult samples, which are expected to be stable, ANS acuity was thus prone to depict substantial heterogeneity.

Such variability seemed to be relative to the generation rule that was followed to create the stimuli (see Figure 2B). The *Panamath* lead to the smallest average value: *w* = 0.18, 95% *CI* [0.15, 0.21]. Studies with any One-dimensional algorithm observed a mean *w* value of 0.20, 95% *CI* [0.17, 0.24]. On the other hand, Multi-dimensional algorithms entailed the largest mean *w* value of 0.29, 95% *CI* [0.18, 0.41]. An unilateral Welch test of equality of means revealed that the algorithm category impacted the mean value of the Weber fraction, *F*_(2, 20.388)_ = 2.768, *p* = 0.043. Pairwise comparison tests (Bonferroni corrected) revealed that the Panamath and the One-dimensional category did not significantly differ from each other, *p* = 0.627; however, Multi-dimensional algorithms were significantly greater than the other two, *p* = 0.024 and *p* = 0.046 respectively for the Panamath and the One-dimensional type. Furthermore, we conducted a Brown-Forsythe test for homogeneity of variance, and this test revealed that variance was statistically different between the algorithms, *F*_(2, 44)_ = 3.965, *p* = 0.026. This confirms that the variability of the values was different between the three categories. In other words, as depicted in Figure 2B, *Panamath* was less variable than the other generation scripts.

Incidentally, in the publications considered in previous analysis, there were 11 explicit reports of Pearson correlation coefficient between the Weber fraction and overall accuracy in the numerical comparison task. Reported coefficient were very high as the mean *r* = 0.97, 95% *CI* [0.96, 0.98], with a minimum *r* of 0.90. This is not surprizing, as Weber fractions are computed from accuracy scores (Piazza et al., 2004; Halberda and Feigenson, 2008). As Inglis and Gilmore (2014) pointed out, such high correlation coefficients question whether the Weber fraction is more informative than general accuracy score, and whether the former should be preferred over the latter.

## Discussion

In the current meta-analysis, we highlighted that the Weber fractions computed from numerical comparison tasks are heterogeneous, even within young adult samples. This variability does not support the view that *w* is a stable parameter devoid of context. As many authors surmised, methodological specificities of the numerical tasks used to compute *w* impacted its value (e.g., Clayton et al., 2015; Smets et al., 2016), and we were able to characterize this substantial heterogeneity in the literature. As depicted in Figures 1, 2, the method used to generate the non-symbolic arrays substantially affected the mean and the variance of the values of *w*. Multi-dimensional algorithms led to larger *w* than the other generation programs. This is likely due to the strategic use–or the unconscious experience–of the non-numerical information that is automatically extracted in the visual cortex during the task (Gebuis et al., 2016; Leibovich et al., 2016). At this point, we want to emphasize that we did not aim for the exhaustive description of all methodological discrepancies that might affect measures of ANS acuity (see Dietrich et al., 2015b). For instance, we did not analyse the impact of the range of the quantities used in each study in their evaluation of *w*. In addition, the current meta-analysis did not provide any theoretical evidence that the ANS does not exist (Leibovich et al., 2016; see alternative view from Gebuis et al., 2016). Our analysis only provides evidence that *w* is not an invariable measure, which may explain some substantial parts in the relation (or non-relation) between ANS acuity and math ability (e.g., Price et al., 2012; Gilmore et al., 2013; Sasanguie et al., 2013).

Interestingly, studies that specifically used the *Panamath* reported homogeneous results. This suggests that the slight methodological difference that we did not consider between these studies–such as the stimulus duration or the numerical range of the arrays–did not drastically impact the measurement of *w*. In other words, *Panamath* studies were thus robust to small dissimilarities in evaluating *w*. It should be noted that our analysis does not simply imply that the *Panamath* reliably assess ANS acuity (see Gebuis et al., 2016; for more detailed methodological considerations). Some might claim that studies from the same laboratory or that use the same exact paradigm arguably tend to show overall higher consistencies, independently from the nature of the task. That being said, our meta-analysis supports that it is possible to reliably measure the same cognitive process in similar numerical comparison tasks, which is reassuring for the literature. It is indeed essential to ascertain that different studies are assessing the same cognitive process before drawing further conclusions about ANS acuity and math ability (see Maxwell et al., 2015; for further considerations about the relevance of replication studies).

Finally, in line with Inglis and Gilmore, 2014 observation, *w* strongly correlated with general accuracy in the literature. It is unsurprising, as *w* indexes *in fine* participant accuracy throughout a numerical task. Yet accuracy is modulated by the way non-numerical visual cues are manipulated, with lower performance when multiple visual dimensions are manipulated at the same time (Smets et al., 2015, 2016). The Weber fraction thus does not provide any additional information about performance than overall accuracy does, mostly when taking a correlational perspective. As Inglis and Gilmore (2014) emphasized, one may wonder whether we should compute *w* at all in the future. With the exception of precise psychophysiological modeling of datasets to highlight specific contribution of numerical and non-numerical dimensions on human behavior (as in DeWind et al., 2015), we believe that most researchers and most clinicians should not bother computing *w*. On the contrary, emphasizing *w* might give the false impression of its invariability, which might incorrectly encourage direct comparison of very different datasets, whereas reporting percentages of correct responses would not favor such direct comparison. This is not trivial, as the evaluation and the training of ANS acuity both have a substantial clinical impact in the assessment and the remediation of math disability (Mazzocco et al., 2011; Park and Brannon, 2013). We are thus in disfavor of the systematic use of the Weber fraction and in favor of the consideration of normative accuracy datasets acquired from the exact same numerical comparison task.

In conclusion, the Weber fraction is an appealing measure of numerical discrimination due to its psychophysiological nature. It is a precious tool to precisely model human behavior. However, researchers and clinicians should not be unaware of its heterogeneity and its context-depend essence. The algorithm used to generate the stimulus set within the task substantially affects its value and its variability. This measure is thus not directly transferable from one study to another. Researchers and practitioners should thus be extremely cautious when comparing comparison tasks.

## Author contributions

MG and AV: original idea and revision; MG: data collection, data analysis, and drafting manuscript.

### Conflict of interest statement

The authors declare that the research was conducted in the absence of any commercial or financial relationships that could be construed as a potential conflict of interest.
