# Supplementary material for: Comparing Numerical Comparison Tasks: A Meta-Analysis of the Variability of the Weber Fraction Relative to the Generation Algorithm
Source: Front Psychol. 2018 Sep 11;9:1694. doi: 10.3389/fpsyg.2018.01694 (PMC6142874; doi:10.3389/fpsyg.2018.01694)
Supplement: Supplementary file 1 [file Table_1.docx]

**Table S1 Description the 115 typical samples (from 68 scientific publications) considered in the meta-analysis.**

| **Authors of the document** | **N of the sample** | **Sample  mean age** | **Algorithm  used** | **Value  of w** |
| --- | --- | --- | --- | --- |
| Attout et al. (2017) | 27 | 7.88 | Multi-dimension | 0.33 |
| Attout et al. (2017) | 27 | 7.22 | Multi-dimension | 0.36 |
| Anobile et al. (2014) | 6 | 27 | Multi-dimension | 0.18 |
| Anobile et al. (2014) | 6 | 27 | Multi-dimension | 0.18 |
| Bugden & Ansari (2016) | 15 | 12 | Panamath | 0.17 |
| Cappelletti et al. (2014) | 30 | 65.78 | One-dimension | 0.30 |
| Cappelletti et al. (2014) | 30 | 24.8 | One-dimension | 0.24 |
| Chesney et al. (2015) | 247 | 19 | Panamath | 0.22 |
| Chesney et al. (2015) | 39 | 22 | Panamath | 0.20 |
| Chu et al. (2013) | 34 | 3.9 | Panamath | 0.84 |
| DeWind & Brannon (2012) | 20 | 21 | One-dimension | 0.33 |
| Dietrich et al. (2015) | 61 | 24 | Multi-dimension | 0.60 |
| Dietrich et al. (2016) | 61 | 24.66 | Multi-dimension | 0.61 |
| Fazio et al. (2014) | 53 | 10.72 | Panamath | 0.21 |
| Geary et al. (2015) | 171 | 15 | Panamath | 0.35 |
| Gilmore et al. (2011) | 101 | 23 | One-dimension | 0.43 |
| Gomez et al. (2015) | 20 | 8 | One-dimension | 0.20 |
| Guillaume et al. (2013) | 29 | 20 | One-dimension | 0.16 |
| Guillaume et al. (2013) | 30 | 18 | One-dimension | 0.20 |
| Guillaume et al. (2016) | 78 | 20 | Multi-dimension | 0.31 |
| Halberda & Feigenson (2008) | 16 | 3 | One-dimension | 0.52 |
| Halberda & Feigenson (2008) | 16 | 4 | One-dimension | 0.38 |
| Halberda & Feigenson (2008) | 16 | 5 | One-dimension | 0.22 |
| Halberda & Feigenson (2008) | 16 | 6 | One-dimension | 0.17 |
| Halberda & Feigenson (2008) | 16 | 20 | One-dimension | 0.10 |
| Halberda et al. (2008) | 64 | 14 | Panamath | 0.27 |
| Hellgren et al. (2013) | 43 | 6 | One-dimension | 0.17 |
| Inglis & Gilmore (2014) | 49 | 33 | Multi-dimension | 0.25 |
| Inglis & Gilmore (2014) | 56 | 8 | Multi-dimension | 0.51 |
| Jang & Cho (2015) | 56 | 22 | One-dimension | 0.17 |
| Jang & Cho (2015) | 57 | 19 | One-dimension | 0.13 |
| Jones et al. (2014) | 10 | 12 | Multi-dimension | 0.21 |
| Khanum et al. (2016) | 42 | 6.4 | Panamath | 0.18 |
| Khanum et al. (2016) | 72 | 6.4 | Panamath | 0.20 |
| Knops et al. (2014) | 14 | 22 | One-dimension | 0.24 |
| Libertus et al. (2011) | 174 | 4 | One-dimension | 0.64 |
| Libertus et al. (2012) | 120 | 20 | Panamath | 0.17 |
| Libertus et al. (2012) | 61 | 20 | Panamath | 0.19 |
| Libertus et al. (2013) | 204 | 4 | Panamath | 0.64 |
| Libertus et al. (2013) | 144 | 5 | Panamath | 0.42 |
| Libertus et al. (2014) | 13 | 4 | Panamath | 0.50 |
| Libertus et al. (2014) | 13 | 6 | Panamath | 0.25 |
| Libertus et al. (2014) | 12 | 9 | Panamath | 0.20 |
| Libertus et al. (2016) | 51 | 6 | Panamath | 0.25 |
| *Lindskog* et al. (2013) | 40 | 24 | One-dimension | 0.23 |
| *Lindskog* et al. (2014) | 100 | 22 | One-dimension | 0.25 |
| Lindskog et al. (2016) | 46 | 22 | One-dimension | 0.25 |
| Liu (2017) | 35 | 10 | Panamath | 0.17 |
| Liu (2017) | 40 | 10.1 | Panamath | 0.20 |
| Lonnemann et al. (2013) | 67 | 7.25 | One-dimension | 0.35 |
| Lonnemann et al. (2013) | 67 | 8.16 | One-dimension | 0.25 |
| Lourenco et al. (2012) | 65 | 19 | One-dimension | 0.39 |
| Lyons & Beilock (2011) | 54 | 20.5 | Multi-dimension | 0.11 |
| Mazzocco et al. (2011) | 37 | 14 | Panamath | 0.26 |
| Mussolin et al. (2012) | 30 | 3.4 | Multi-dimension | 0.51 |
| Mussolin et al. (2012) | 49 | 4.3 | Multi-dimension | 0.37 |
| Mussolin et al. (2012) | 31 | 5.5 | Multi-dimension | 0.25 |
| Mussolin et al. (2012) | 41 | 5.9 | Multi-dimension | 0.18 |
| Mussolin et al. (2014) | 57 | 4 | Multi-dimension | 0.42 |
| Mussolin et al. (2014) | 57 | 4.7 | Multi-dimension | 0.26 |
| Norris & Castronovo (2016) | 32 | 19.8 | One-dimension | 0.28 |
| Norris & Castronovo (2016) | 32 | 20.8 | One-dimension | 0.20 |
| *Norris* et al. (2015) | 26 | 20 | Panamath | 0.18 |
| *Norris* et al. (2015) | 26 | 65 | Panamath | 0.18 |
| Odic (2017) | 24 | 3.33 | One-dimension | 0.98 |
| Odic (2017) | 34 | 4.91 | One-dimension | 0.59 |
| Odic (2017) | 49 | 7.05 | One-dimension | 0.31 |
| Odic (2017) | 47 | 8.95 | One-dimension | 0.21 |
| Odic (2017) | 31 | 11.32 | One-dimension | 0.16 |
| Odic (2017) | 15 | 20.06 | One-dimension | 0.17 |
| Odic et al. (2013a) | 8 | 3 | One-dimension | 0.52 |
| Odic et al. (2013a) | 8 | 4 | One-dimension | 0.46 |
| Odic et al. (2013a) | 8 | 5 | One-dimension | 0.30 |
| Odic et al. (2013a) | 8 | 6 | One-dimension | 0.22 |
| Odic et al. (2013a) | 8 | 19 | One-dimension | 0.13 |
| Odic et al. (2013b) | 40 | 3 | One-dimension | 0.64 |
| Odic et al. (2014) | 10 | 4 | One-dimension | 0.57 |
| Odic et al. (2014) | 10 | 5 | One-dimension | 0.78 |
| Odic et al. (2014) | 10 | 5 | One-dimension | 0.52 |
| Odic & Halberda (2015) | 12 | 20 | One-dimension | 0.13 |
| Oliveira et al. (2014) | 40 | 10 | One-dimension | 0.24 |
| Olsson et al. (2016) | 48 | 9 | Panamath | 0.18 |
| Park & Starns (2015) | 120 | 20 | One-dimension | 0.16 |
| Patalano et al. (2015) | 57 | 20 | Panamath | 0.15 |
| Piazza et al. (2010) | 29 | 10 | One-dimension | 0.25 |
| Piazza et al. (2010) | 20 | 26 | One-dimension | 0.15 |
| Pietroski et al. (2009) | 12 | 20 | One-dimension | 0.32 |
| Pinheiro-Chagas et al. (2014) | 162 | 10 | One-dimension | 0.24 |
| Price et al. (2012) | 39 | 22 | One-dimension | 0.22 |
| Price et al. (2012) | 39 | 22 | One-dimension | 0.38 |
| Shusterman et al. (2016) | 42 | 4.1 | One-dimension | 0.60 |
| Shusterman et al. (2016) | 39 | 4.3 | One-dimension | 0.54 |
| Skagerlund & Träff (2014) | 133 | 9.69 | Panamath | 0.51 |
| Skagerlund & Träff (2016) | 32 | 10 | Panamath | 0.26 |
| Skagerlund & Träff (2016) | 31 | 8 | Panamath | 0.49 |
| Smets et al. (2014) | 22 | 19 | Multi-dimension | 0.37 |
| Smets et al. (2015) | 26 | 18 | Multi-dimension | 0.28 |
| Smets et al. (2015) | 26 | 18 | One-dimension | 0.11 |
| Smets et al. (2015) | 26 | 18 | Multi-dimension | 0.09 |
| Smets et al. (2016) | 40 | 21 | Multi-dimension | 0.25 |
| Smets et al. (2016) | 40 | 21 | One-dimension | 0.15 |
| Starr et al. (2013) | 48 | 3.6 | One-dimension | 0.46 |
| Szücs et al. (2013) | 20 | 7.5 | Multi-dimension | 0.77 |
| Szücs et al. (2013) | 22 | 25.7 | Multi-dimension | 0.30 |
| Tinelli et al. (2015) | 26 | 8 | Multi-dimension | 0.27 |
| Tosto et al. (2014) | 2258 | 16.6 | One-dimension | 0.27 |
| vanMarle et al. (2014) | 155 | 3 | Panamath | 0.50 |
| van den Berg et al. (2017) | 30 | 26.1 | One-dimension | 0.13 |
| van den Berg et al. (2017) | 30 | 26.1 | One-dimension | 0.11 |
| van den Berg et al. (2017) | 85 | 25.2 | One-dimension | 0.16 |
| van den Berg et al. (2017) | 85 | 25.2 | One-dimension | 0.15 |
| van den Berg et al. (2017) | 85 | 25.2 | One-dimension | 0.16 |
| Winman et al. (2014) | 213 | 40 | One-dimension | 0.25 |
| Wong et al. (2014) | 154 | 6 | One-dimension | 0.23 |
| Wong et al. (2016) | 179 | 6 | One-dimension | 0.26 |

**Note. Odic et al. (2013a) is Odic, Libertus, Feigenson, & Halberda (2013); Odic et al. (2013b) is Odic, Pietroski, Hunter, Lidz, & Halberda (2013)**
